# Differential Contribution of Constituent Metal Ions to the Cytotoxic Effects of Fast-Dissolving Metal-Oxide Nanoparticles

**DOI:** 10.3389/fphar.2018.00015

**Published:** 2018-01-22

**Authors:** Jiyoung Jeong, Sung-Hyun Kim, Seonghan Lee, Dong-Keon Lee, Youngju Han, Soyeon Jeon, Wan-Seob Cho

**Affiliations:** Laboratory of Toxicology, Department of Medicinal Biotechnology, College of Health Sciences, Dong-A University, Busan, South Korea

**Keywords:** A549, cytotoxicity, dose-response, fast-dissolving nanoparticle, THP-1, uptake

## Abstract

The main mechanism of toxicity for fast-dissolving nanoparticles (NPs) is relatively simple as it originates from the intrinsic toxicity of their constituent elements rather than complicated surface reactivity. However, there is little information about the compared toxicity of fast-dissolving NP and its constituent ion, which is essential for understanding the mechanism of NP toxicity and the development of a structure-toxicity relationship (STR) model. Herein, we selected three types of fast-dissolving metal-oxide NPs (CoO, CuO, and ZnO) and constituent metal chlorides (CoCl_2_, CuCl_2_, and ZnCl_2_) to compare dose-response curves between NP and its constituent metal. These materials were treated relevant cell lines for inhalation setting (i.e., differentiated THP-1 cells for macrophages and A549 cells for alveolar epithelial cells) and cytotoxicity as an endpoint was evaluated at 24 h post-incubation. The results showed that CoO and CuO NPs in both cell types showed similar patterns of dose-response curves and cytotoxic potential compared to that of their respective metal chloride. On the other hand, ZnO NPs in both cell types showed a completely different dose-response curve compared to that of ZnCl_2_: ZnO NPs showed modest slope and much less potential for cytotoxicity compared to that of ZnCl_2_. These results imply that fast-dissolving metal-oxide NPs are not always have similar dose-response curves and toxic potentials compared to their constituent metal chlorides and this may be due to the differential mechanism of intracellular uptake of these substances and their interaction with intracellular detoxification molecules. Further investigations are needed for the use of toxic potential of metal ions as a predicting factors of fast-dissolving NPs toxicity. In addition, chelating agent specific for dissolved metal ions can be applied for the treatment of these fast-dissolving NPs.

## Introduction

Metal-oxide nanoparticle (NP) is one of the major types of nanomaterials, which is used for industrial as well as biomedical applications. As the number and production volume of NPs has increased, so have concerns about their toxicity exponentially increased in recent years. Inhalation, compared to other routes of exposure, has a much higher risk to humans because of the high deposition rate in the alveoli, which have limited clearance mechanisms. Thus, inhalation of metal-oxide NPs can cause various types of acute and chronic lung injuries (Oberdorster et al., 2005; Cho et al., 2010). With exponential increases in the types of NPs and safety concerns associated with them, there is an urgent need for the development of prescreening tools for NP safety using a structure-toxicity relationship (STR) model (Puzyn et al., 2009). The first step for a STR model is qualitative and quantitative evaluation of the structural parameters related with the toxicity of a NP.

Among various physicochemical properties of NPs, biopersistence or durability is one of the key determinants of its toxic potential (Donaldson et al., 2013; Braakhuis et al., 2014). NPs in most cases are minimally dissolving under normal physiological conditions but some NPs have shown to be dissolving to a significant degree when dispersed in acidic physiological conditions such as gastric fluid and lysosomal fluid (Cho et al., 2012b, 2013b). Although more studies are needed for defining the degrees of dissolution, previous studies including our own have shown that CoO, CuO, and ZnO NPs were almost completely dissolved within 24 h under acidic physiological conditions. These fast-dissolving NPs were ranked in a high-toxicity group compared to the less soluble NPs such as CeO_2_, Co_3_O_4_, Cr_2_O_3_, NiO, and TiO_2_ (Rushton et al., 2010; Cho et al., 2012b; Jeong et al., 2015; Liu et al., 2015). In addition, our previous studies have shown that the toxicity of fast-dissolving metal-oxide NPs was closely related with the intrinsic toxic potential of its constituent metal ions (Cho et al., 2012b, 2013a). Although the central mechanism of toxicity for fast-dissolving NPs is the intrinsic toxicity of its constituent elements rather than surface reactivity, information comparing the toxic potential of fast-dissolving NPs and its constituent ion is lacking. This knowledge is essential for understanding the mechanism of toxicity and the development of prescreening tools for fast-dissolving NPs.

The comparison of *in vivo* toxic potential of NP and its constituent metal ion may be more useful than *in vitro* assays. However, the direct use of the comparative *in vivo* toxicity data between NPs and its constituent metal ion would be complicated by several limitations such as differences in deposition site (i.e., metal ions usually deposit in the upper respiratory tract in the inhalation setting, while NPs have high deposition rate in the lower respiratory tract or alveoli) and extrapulmonary translocation (i.e., metal ions have higher extrapulmonary translocation than NPs). In this regard, the *in vitro* comparison of toxic potential using relevant cell lines under inhalation condition can overcome these limitations and provide important information for the STR modeling of fast-dissolving NPs. Herein, we investigated the differential cytotoxicity and levels of pro-inflammatory cytokines between fast-dissolving NPs and their constituent metal ions using relevant cell lines for inhalation settings: macrophages-like (differentiated) THP-1 cells and alveolar epithelial cell-like A549 cells.

## Materials and Methods

### Physicochemical Characterization of NPs and Metal Chlorides

Based on our previous studies, CoO NP (NanoAmor, Houston, TX, United States), CuO NP (Sigma–Aldrich, St. Louis, MO, United States), and ZnO NP (NanoScale Corporation, Manhattan, KS, United States) were selected as representative fast-dissolving high-toxicity NPs, while Co_3_O_4_ NP (NanoAmor) and TiO_2_ NP (NanoAmor) were selected as representative low-solubility high-toxicity NP and low-solubility low-toxicity NP, respectively (Cho et al., 2010; Jeong et al., 2015). Metal chlorides (CoCl_2_, CuCl_2_, and ZnCl_2_; Sigma–Aldrich) were used for the constituent metal ions. The primary size of NPs was measured using a transmission electron microscope (TEM; JEOL, Tokyo, Japan) and the hydrodynamic size of NPs dispersed in various media including distilled water (DW) and cell culture media was measured using a Zetasizer-Nano ZS (Malvern, Malvern Hills, United Kingdom), as previously described (Jeong et al., 2015). The surface areas of NPs were measured using Micromeritics Tristar 3000 analyzer (Micromeritics, Ltd., Bedfordshire, United Kingdom) for CuO and ZnO, or ASAP 2420 (Micromeritics, Ltd.) for CoO. The Brunauer–Emmett–Teller (BET) method was used to calculate the surface area for both Micromeritics instruments. The levels of endotoxin contamination were measured at 500 μg/mL of NP in PBS, the highest dose used in this study, using an endpoint chromogenic *Limulus* amebocyte lysate (LAL) assay kit (Cambrex, Walkersville, MD, United States).

### Dissolution Assay for NPs

The dissolution of NPs was measured in artificial lysosomal fluid (pH 5.5) or PBS (pH 7.4) as previously described (Jeong et al., 2015). Briefly, NPs were dispersed in each medium at 100 μg/mL and incubated for 24 h at room temperature with continuous agitation. Then, three rounds of centrifugation at 15000 × *g* for 30 min were performed to collect the NP-free supernatant and the absence of NP was confirmed by dynamic light scattering analysis using a Zetasizer-Nano ZS (Malvern). The concentration of metal ions in the supernatant was measured using inductively coupled plasma-optical emission spectrometry (ICP-OES) (Optima 8300, PerkinElmer, Shelton, CT, United States) by the Center for Collaborative Instruments at Dong-A University. The dissolution was calculated as the percentage of dissolved metal concentration compared to the initial mass of metal in the NP dispersion.

### Preparation of NPs and Metal Chlorides for Cell Culture

Because NP showed agglomerations in culture medium as shown in **Table 1**, it was pre-dispersed with fetal bovine serum (FBS) as described in our previous study (Cho et al., 2013a). Briefly, the stock solution of NP was dispersed in DW at 5000 μg/mL and sonicated with 28 KHz operation frequencies and 400 W output power for 5 min in a bath sonicator (Saehan-Sonic, Seoul, South Korea). Then FBS was added 5% (for A549 cells) or 10% (for THP-1 cells) of working volume and sonicated with bath sonicator (Saehan-Sonic) for 5 min. FBS-free culture medium was then added for working concentrations (0–500 μg/mL). Because metal chlorides showed precipitation in Dulbecco’s Modified Eagle’s Medium (DMEM; GIBCO, Grand Island, NY, United States), phosphate-free DMEM (GIBCO), which showed no precipitation, was used as the culture medium for metal chlorides.

**Table 1:** Physicochemical properties of NPs.

NPs	CoO	CuO	ZnO	Co_3_O_4_	TiO_2_
Primary size (nm)	75.0 ± 2.4	28.5 ± 0.7	16.5 ± 0.4	33.8 ± 2.1	39.9 ± 1.4
Surface area (m^2^g)	8.5	29	48.2	35.8	27.5
**Hydrodynamic size (nm) in**					
DW	493 ± 10	176 ± 1.5	592 ± 21	153 ± 3.5	472 ± 10
RPMI-1640	116 ± 13	192 ± 4.6	296 ± 14	164 ± 3.9	517 ± 14
DMEM	335 ± 57	188 ± 5.3	491 ± 51	170 ± 6.2	527 ± 25
**Polydispersity in**					
DW	0.35 ± 0.01	0.19 ± 0.02	0.55 ± 0.04	0.20 ± 0.02	0.41 ± 0.03
RPMI-1640	0.38 ± 0.02	0.18 ± 0.02	0.58 ± 0.02	0.22 ± 0.01	0.53 ± 0.06
DMEM	0.76 ± 0.01	0.18 ± 0.00	0.81 ± 0.04	0.21 ± 0.01	0.35 ± 0.01
**Zeta potential (mV) in**					
DW	22.27 ± 0.20	38.80 ± 0.90	–8.74 ± 1.57	26.40 ± 0.42	19.30 ± 0.50
RPMI-1640	–21.40 ± 0.52	–21.33 ± 0.88	–22.30 ± 0.49	–20.67 ± 0.69	–23.40 ± 0.53
DMEM	–21.50 ± 0.52	–21.17 ± 0.54	–20.57 ± 0.75	–20.07 ± 0.52	–22.50 ± 0.55
Endotoxin (U/mL)	<0.1	<0.1	<0.1	<0.1	<0.1
Solubility (%) in ALF	95.27	100	99.06	11.46	0
Solubility (%) in PBS	1.34	1.71	2.82	0.02	0

*DW, distilled water; ALF, artificial lysosomal fluid; PBS, phosphate-buffered saline.*

### Cell Culture

Because the primary target cell for inhaled particle is alveolar macrophages and activated macrophages by NPs can damage alveolar epithelial cells by releasing various inflammatory mediators and cytotoxic materials, we selected relevant cell lines for inhalation settings as macrophage-like (differentiated) THP-1 cells (American Type Culture Collection, Manassas, VA, United States) and alveolar epithelial cell-like A549 cells (European Collection of Animal Cell Cultures, Salisbury, United Kingdom). Both cell lines were cultured and maintained as described in our previous study (Cho et al., 2013a).

### Treatment of Cells with NPs or Metal Chlorides and Evaluation of Cytotoxicity

Because THP-1 cells are monocytic cells, they differentiated to macrophage-like cells with phorbol myristate acetate (PMA; Sigma–Aldrich) treatment. Briefly, differentiated THP-1 cells were prepared by seeding at 5 × 10^5^ cells/mL with 10 ng/mL of PMA in 96-well plates and culturing for 2 days. A549 cells were seeded at 2 × 10^5^ cells/mL in 96-well plates and incubated overnight for 80% confluency. THP-1 cells or A549 cells were then washed three times with pre-warmed PBS and were treated with NPs dispersed in culture medium at various concentrations from 0 to 500 μg/mL. After 24 h incubation, the supernatant was collected for measurement of pro-inflammatory cytokines, and cells were washed three times with pre-warmed PBS to avoid or minimize interference on the cytotoxicity assay by NPs or dissolved metal ions (Rosslein et al., 2015). Then, 100 μL of fresh complete medium was added with 20 μL of 3-(4,5-dimethylthiazol-2-yl)-5- (3-carboxymethoxyphenyl)-2-(4-sulfophenyl)-2H-tetrazolium (MTS) solution (Promega, Madison, WI, United States). After 2 h incubation at 37°C in a CO_2_ incubator, the supernatant was transferred to clean plates to minimize colorimetric interference by cells containing NPs and absorbance was measured at 490 nm. To compare the dose-response and potential of cytotoxicity between fast-dissolving NPs and its respective metal ions, the masses of treatment doses were converted into molar concentrations.

### Measurement of Levels of Pro-inflammatory Cytokines

The pro-inflammatory cytokines levels in the cell-culture supernatant were measured using an enzyme-linked immunosorbent assay (ELISA) kit. In differentiated THP-1 cells, interleukin-1β (IL-1β) was measured to evaluate the effect of NPs on the activation of the inflammasome (Yazdi et al., 2010). On the other hand, IL-8 was measured in A549 cells to evaluate the role of NPs and metal chlorides on the stimulation of lung epithelial cells. Both ELISA kits were purchased from the R&D systems (Duoset kit, Minneapolis, MN, United States). Based on the cell viability data, more than three doses were selected for the measurement of IL-1β in THP-1 cells, the selected doses were 5, 10, and 20 μg/mL for fast-dissolving NPs and metal chlorides; and 25, 50, and 100 μg/mL for control NPs (Co_3_O_4_ and TiO_2_). Likewise, for A549 cells, the selected doses were 1–50 μg/mL for fast-dissolving NPs and metal chlorides; 25, 50, and 100 μg/mL for control NPs (Co_3_O_4_ and TiO_2_). The mass doses were converted into the molar concentrations.

### Treatment of Cells with Chelated Dissolved Metal Ions

To evaluate the role of dissolved metal ions in the cytotoxicity, cells were treated with solubilized ions followed by chelation, as described in our previous study with slight modification (Cho et al., 2012a). Briefly, the stock solution of metal chlorides at 5 mg/mL in DW were incubated with 50 mg/mL Chelex 100 beads (Sigma–Aldrich) and mixed for 4 h at room temperature with continuous agitation. The beads were then removed by centrifugation at 15000 × *g* for 15 min. The collected supernatant was diluted to the final working concentration (100 μg/mL) with cell culture medium and treated to A549 or differentiated THP-1 cells.

### Statistical Analysis

Data were expressed as mean ± SEM (*n* = 8). We analyzed statistical differences using one-way analysis of variance (ANOVA) followed by *post hoc* Tukey’s pairwise comparisons using a GraphPad Prism V6.0 (GraphPad Software, San Diego, CA, United States). A *p* value less than 0.05 was considered statistically significant.

## Results

### Physicochemical Properties of the NPs

**FIGURE 1:**
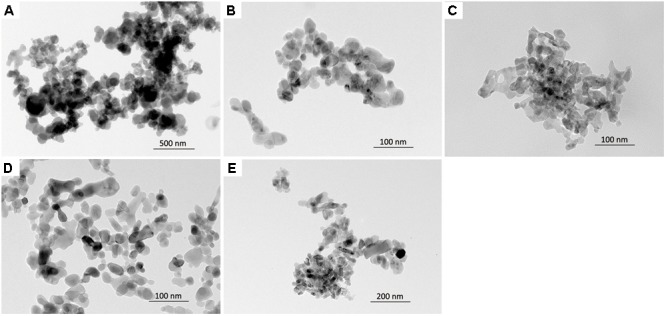
The morphology of NPs observed by a transmission electron microscopy. **(A)** CoO NP, **(B)** CuO NP, **(C)** ZnO NP, **(D)** Co_3_O_4_ NP, and **(E)** TiO_2_ NP.

The physicochemical properties of NPs are presented in **Table 1**. All NPs were less than 100 nm in size, as measured by TEM (**Figure 1**). The hydrodynamic size of NPs in DW showed that all types of NPs were agglomerated with a size range 153–592 nm: Co_3_O_4_ NP showed the least agglomeration, while the ZnO NP showed the most. The hydrodynamic size of NPs in the culture medium showed that the pre-dispersion of NPs using FBS significantly reduced agglomeration, which resulted in size ranges improved or comparable to those in DW. The surface charge measured by zeta potential showed that ZnO NP in DW was negative, while the other NPs were positive. However, the zeta potential of NPs in culture medium showed that all types of NPs had negative zeta potential with similar size ranges about -20 mV. The LAL assay showed that all NPs had endotoxin levels less than the limit of detection (LOD; 0.1 U/mL). The dissolution study showed that incubation of fast-dissolving NPs (CoO, CuO, and ZnO) in artificial lysosomal fluid (pH 5.5) for 24 h resulted dissolution more than 95%, while Co_3_O_4_ and TiO_2_ NPs had 11.46 and 0% dissolution, respectively. However, all NPs dispersed in PBS showed less than 3% dissolution.

### Differential Cytotoxic Potential between NPs and Metal Chlorides in Differentiated THP-1 Cells

**FIGURE 2:**
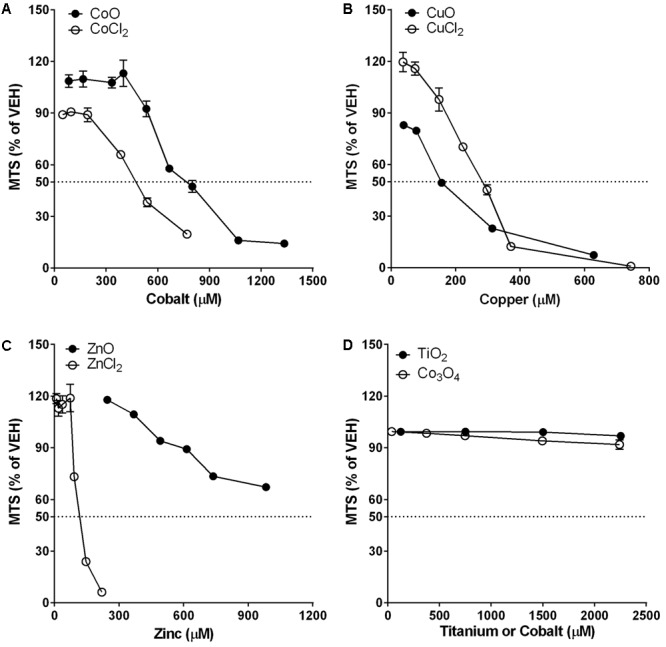
Comparison of dose-response curves of metal-oxide NPs and their respective constituent metal chlorides in differentiated THP-1 cells. Cytotoxicity was measured by MTS assay. Note that the mass doses were converted into molar concentrations. **(A)** CoO NP showed a similar trend in the dose-response curve but less cytotoxic potential than CoCl_2_. **(B)** CuO NP showed a similar trend in the dose-response curve but higher cytotoxic potential than CuCl_2_. **(C)** ZnO NP showed a completely different trend in the dose-response curve with much less cytotoxic potential than ZnCl_2_. **(D)** Biopersistent TiO_2_ and Co_3_O_4_ NPs showed no cytotoxicity within the tested doses (<180 μg/mL; 2254 μM for TiO_2_ and 2243 μM for Co_3_O_4_). Values are mean ± SEM from eight independent experiments.

The dose-response curves of NPs and metal chlorides are presented in **Figure 2**. The dose-response curves of CoO NP vs. CoCl_2_ and CuO NP vs. CuCl_2_ on comparison showed similar trends but slightly different cytotoxic potential. CoO NP showed lower cytotoxic potential than CoCl_2_, while CuO NP showed higher cytotoxic potential than CuCl_2_. The EC_50_ value for CoO NP and CoCl_2_ was 775 μM (58 μg/mL) and 475 μM (62 μg/mL), respectively. The EC_50_ value of CuO NP and CuCl_2_ was 155 μM (12 μg/mL) and 284 μM (38 μg/mL), respectively. Unlike CoO and CuO NPs, ZnO NP showed a completely different trend of the dose-response curve when compared to ZnCl_2_. The cytotoxic potential of ZnO NP was much less than that of ZnCl_2_. The EC_50_ value of ZnO NP was higher than 983 μM (80 μg/mL), while EC_50_ value of ZnCl_2_ was 120 μM (16 μg/mL). Biopersistent TiO_2_ and Co_3_O_4_ NPs showed no cytotoxicity within the tested doses.

### Differential IL-1β Concentrations between Differentiated THP-1 Cells Treated with NPs and Metal Chlorides

**FIGURE 3:**
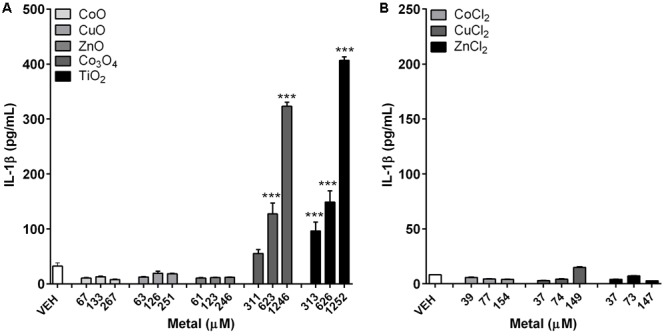
Concentrations of IL-1β in differentiated THP-1 cells after treatment with metal-oxide NPs or metal chlorides. **(A)** Fast-dissolving NPs showed no significant increase, while biopersistent Co_3_O_4_ and TiO_2_ NPs showed significant increase in a dose-dependent manner. **(B)** Metal chlorides showed no significant increase. The selected doses were 5, 10, and 20 μg/mL for fast-dissolving NPs and metal chlorides; and 25, 50, and 100 μg/mL for biopersistent control NPs (Co_3_O_4_ and TiO_2_). Note that the doses were converted into molar concentrations. Values are mean ± SEM from eight independent experiments. ^∗∗∗^*p* < 0.001 vs. vehicle control (VEH).

IL-1β levels showed no significant changes on treatment with either fast-dissolving NPs (CoO, CuO, and ZnO) or metal chlorides (CoCl_2_, CuCl_2_, and ZnCl_2_) (**Figure 3**). However, the biopersistent control NPs, both Co_3_O_4_ and TiO_2_, showed significant increase in IL-1β levels in a dose-dependent manner (**Figure 3A**).

### Differential Cytotoxic Potential between NPs and Metal Chlorides in A549 Cells

**FIGURE 4:**
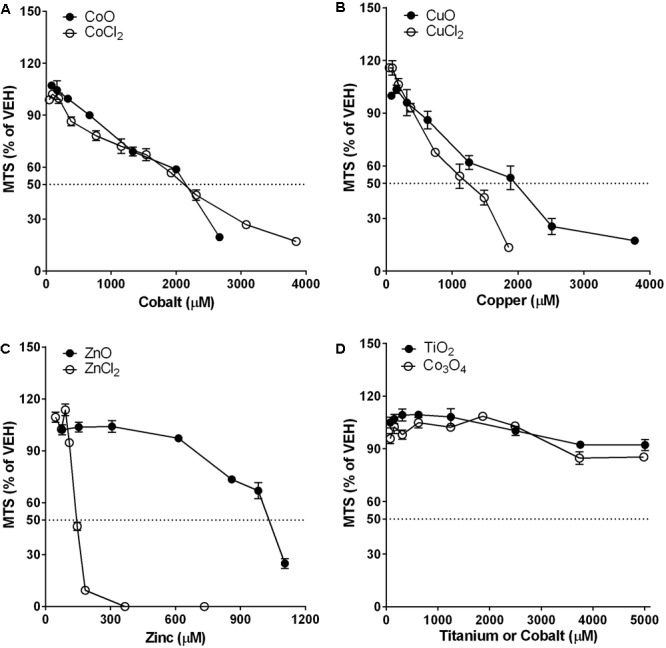
Comparison of dose-response curves of metal-oxide NPs and their respective constituent metal chlorides in A549 cells. Cytotoxicity was measured by MTS assay. Note that the mass doses were converted into molar concentrations. **(A)** CoO NP showed a similar trend in the dose-response curve and almost identical cytotoxic potential compared to CoCl_2_. **(B)** CuO NP showed a similar trend in the dose-response curve but less cytotoxic potential than CuCl_2_. **(C)** ZnO NP showed a completely different trend in the dose-response curve with much less cytotoxic potential than ZnCl_2_. **(D)** Biopersistent TiO_2_ and Co_3_O_4_ NPs showed no cytotoxicity within the tested doses (<400 μg/mL; 5008 μM for TiO_2_ and 4983 μM for Co_3_O_4_). Values are mean ± SEM from eight independent experiments.

Cytotoxicity measured by the MTS assay in A549 cells is presented in **Figure 4**. Dose-response curve of CoO NP was overlapped with that of CoCl_2_, and the EC_50_ value for both CoO NP and CoCl_2_ was 2150 μM (161 μg/mL for CoO NPs and 279 μg/mL for CoCl_2_). While, CuO NP and CuCl_2_ showed similar trends in their dose-response curves, the cytotoxic potential of CuO NP was slightly less than that of CuCl_2_. The EC_50_ values for CuO NP and CuCl_2_ were 1960 μM (156 μg/mL) and 1235 μM (166 μg/mL), respectively. Unlike CoO and CuO NP, the dose-response curve of ZnO NP was completely different compared to that of ZnCl_2_. The EC_50_ values of ZnO and ZnCl_2_ in A549 cells were 1030 μM (84 μg/mL) and 143 μM (20 μg/mL), respectively. Biopersistent TiO_2_ and Co_3_O_4_ NPs showed no significant cytotoxicity within the tested doses.

### Differential IL-8 Concentrations between A549 Cells Treated with NPs and Metal Chlorides

**FIGURE 5:**
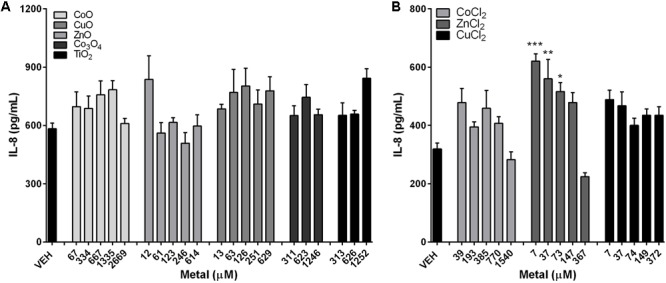
Concentrations of IL-8 expression in the A549 cells after treatment with metal-oxide NPs or metal chlorides. IL-8 levels in A549 cells after treatment with **(A)** metal-oxide NPs, and **(B)** metal chlorides. NPs were treated at various doses ranging 1–200 μg/mL. Note that the mass doses were converted into molar concentrations. Values are mean ± SEM from eight independent experiments. ^∗^*p* < 0.05, ^∗∗^*p* < 0.01, and ^∗∗∗^*p* < 0.001 vs. VEH.

IL-8 levels showed no significant changes on treatment with either fast-dissolving NPs (CoO, CuO, and ZnO) or biopersistent control NPs (Co_3_O_4_ and TiO_2_) (**Figure 5**). Treatment of metal chlorides (CoCl_2_, CuCl_2_, and ZnCl_2_) also showed no biologically meaningful changes in the IL-8 levels, although ZnCl_2_ showed slight increases in the low and mid dose (**Figure 5**).

### Cytotoxicity of Differentiated THP-1 Cells and A549 Cells after Chelation of Metal Chlorides

**FIGURE 6:**
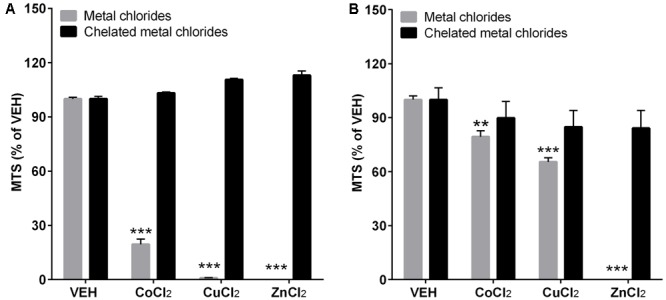
The cytotoxicity assay after chelation of metal chlorides. The differentiated THP-1 cells **(A)** and A549 cells **(B)** were treated with or without chelation of metal ions. To chelated metal ions, Chelex 100 beads (Sigma–Aldrich) were applied to the stock solution of metal chlorides in DW and diluted to the final working concentration in cell culture media. The treated dose for both metal chlorides and chelated metal chlorides was 100 μg/mL. Cytotoxicity was measured by MTS assay. Values are mean ± SEM from eight independent experiments. ^∗∗^*p* < 0.01, and ^∗∗∗^*p* < 0.001 vs. VEH.

The chelation of metal chlorides showed dramatic recovery of cytotoxicity compared to non-chelated metal chlorides (**Figure 6**). Although metal chlorides at 100 μg/mL showed significant cytotoxicity in both cell lines, chelation of metals did not show any cytotoxicity at the tested dose.

## Discussion

Non-biopersistent fast-dissolving metal-oxide NPs such as CoO, CuO, and ZnO are generally ranked in a higher toxicity group than biopersistent metal-oxide NPs such as CeO_2_, Co_3_O_4_, Cr_2_O_3_, NiO, and TiO_2_ because of the wide-spread to target cells and fast dissolution in the acidic lysosomal fluid like a Trojan-horse (Cho et al., 2010; Rushton et al., 2010; Liu et al., 2015). Because one of the main mechanisms of toxicity for fast-dissolving NPs is the loss in durability by the acidic lysosomal fluid inside phagolysosomes, prediction of their toxicity might be possible using the intrinsic toxic potential of their constituent ions. However, there is little known about the correspondence between the toxic potential of fast-dissolving metal-oxide NP and its constituent metal. Thus, we believe that evaluation of the differential toxic effect of NP and metal ion using an *in vitro* system which can mimic *in vivo* inhalation settings might provide better information for the mechanism of toxicity for fast-dissolving metal oxide NPs. Herein, we found that the cytotoxic potential as well as pattern of dose-response curves of fast-dissolving NPs (CoO, CuO, and ZnO) in macrophage-like THP-1 cells or alveolar epithelial cell-like A549 cells had both similarities and differences when compared to their respective constituent ions, and responses also slightly differed by the types of tested cells.

In this study, we evaluated cytotoxicity by the MTS assay, which measures mitochondrial activity. Colorimetric or fluorometric assays can be sensitive to interference by NPs or dissolved metal ions (Kroll et al., 2012). However, we minimized this interference by washing away any NPs or metal ions dispersed in culture medium before adding the MTS solution. In addition, NPs inside cells can be further excluded by collecting the cell-free supernatant prior to measure the absorbance. This modified MTS assay has many advantages compared to other methods such as microscopically observing stained cells (e.g., trypan blue exclusion assay, calcein acetoxymethyl ester staining, and neutral red staining), lactate dehydrogenase (LDH) assay, and flow cytometry. Counting dead or live cells after staining cells using a microscope can be inaccurate because NPs (e.g., black particles) can physically interfere in distinguishing colors, and omission of some dead cells due to detachment from the culture plate (Darolles et al., 2013). LDH assay can be interfered by NPs (by adsorption of formazan) or dissolved ions (by inhibition of the enzymatic reaction), which are hard to exclude in the cell culture supernatants (Holder et al., 2012). Thus, the modified MTS assay used in this study is easy and fast to perform and provides reliable results. Other methods similar to the MTS assay are Alamar Blue assay, water-soluble tetrazolium salt-1 (WST-1), Cell Counting Kit-8 (CCK-8), and 2,3-*bis*-(2-methoxy-4-nitro-5-sulfophenyl)-2H-tetrazolium-5-carboxanilide (XTT) assay.

The comparison of dose-responses between NP and its constituent metal ions showed similar and differential effects in both cell lines. The similar effects of CoO and CoCl_2_ were consistent with our previous study, where intratracheal instillation of CoO NP showed similar or slightly less inflammogenic and cytotoxic potential than CoCl_2_ at the same molar concentration (Jeong et al., 2015). The role of dissolved ions in the toxicity of fast-dissolving NPs was further supported by a previous study which showed that less soluble micro-scale CuO particles had much less cytotoxicity than highly soluble nano-scale CuO NPs, and highly soluble nano-scale CuO NPs in A549 cells and HeLa S3 cells showed similar dose-response curves of cytotoxicity compared to CuCl_2_ at the same molar concentration (Semisch et al., 2014). The differential cytotoxic potential of NPs compared to its constituent metal chlorides by the types of cells found in this study might be due to the difference in the activity of cellular uptake as well as in the sensitivity of cell types. Endocytosis and release of dissolved metal ions inside phagolysosomes is the main toxicity mechanism of fast-dissolving NPs, while metal ions can enter cells mostly via transporters, thus fast-dissolving NPs need longer time for dissolution but has much higher intracellular metal concentrations than those of soluble ions (Colognato et al., 2008; Ponti et al., 2009; Semisch et al., 2014). Taking into account variation in the mechanism of cellular uptake and the resultant difference in cellular localization and concentration, the direct comparison of fast-dissolving NPs against their constituent ions might be limited.

Dissolved free-zinc from either ZnO NP or ZnCl_2_ induces apoptosis or necrosis in endothelial and epithelial cells by damaging mitochondrial function followed by elevation of intracellular reactive-oxygen species (Wiseman et al., 2006; Kim et al., 2010). However, in this study, ZnO NP in comparison to ZnCl_2_ showed very low toxic potential with completely different trend of dose-response curves in both cell types. The lesser cytotoxicity of ZnO NP in comparison to ZnCl_2_ was also reported in Daphnia (Adam et al., 2014; Garcia-Gomez et al., 2014) and in rats (Cho et al., 2011; Amara et al., 2014).

**FIGURE 7:**
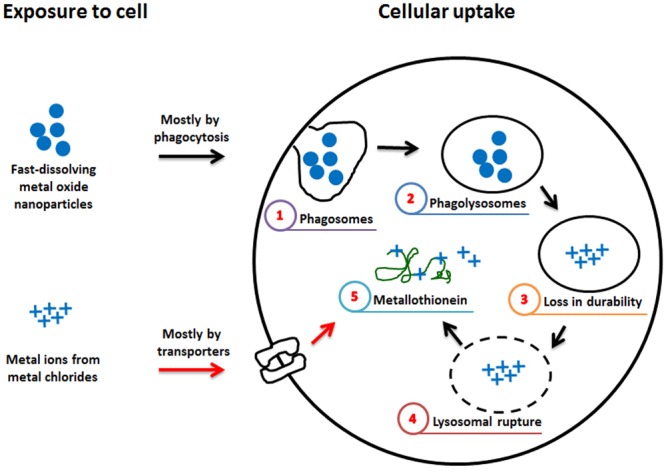
Hypothetical diagram for the differential effect of fast-dissolving metal-oxide NPs and their respective metal chlorides. The differential cytotoxic effect of fast-dissolving metal-oxide NPs and metal ions might be due to the difference in the mechanism of intracellular uptake. NP enters mostly by phagocytosis and (1) phagosomes can fuse with lysosomes to form (2) phagolysosomes. In the acidic lysosomal fluid, fast-dissolving NP can be (3) susceptible to breakdown and its toxic metal ions can (4) rupture the lysosomal membrane. (5) In the cytosol, some of the released metal ions bind with metallothionein, while metal ions outside the cell enter mostly via transporters and (5) bind with metallothionein. The reduced cytotoxicity of ZnO compared to ZnCl_2_ might be due to Zn-metallothionein that can act as anti-oxidant, while the comparable cytotoxicity of CoO vs. CoCl_2_ and CuO vs. CuCl_2_ might be due to Cu-metallothionein that can act as pro-oxidant, although the effect of Co-metallothionein is not known (Hahn et al., 2001).

The differential toxic effects between ZnO NP and ZnCl_2_ might be due to differences in cellular uptake mechanisms and the microenvironment they encounter (**Figure 7**). The breakdown of ZnO NP is initiated when it enters into acidic phagolysosomes, while ionized zinc from ZnCl_2_ in culture medium can enter cells immediately after treatment, mostly via specific transporters (Cho et al., 2013a). Furthermore, the free zinc localized in the cytosol stimulates metallothionein expression, and NPs have a greater potential for the stimulation of metallothionein than their component metal ion (Hahn et al., 2001; Cuillel et al., 2014; Bulcke and Dringen, 2015). Metallothionein-zinc complex can mitigate the toxicity of free-zinc, which is very toxic because of its oxidation ability. However, copper can replace zinc from the metallothionein-zinc complex, thus the copper-metallothionein complex is a pro-oxidant, while the zinc-metallothionein complex is an anti-oxidant (Hahn et al., 2001). The variable role of metallothionein depending on the type of metal might explain why ZnO NP has different cytotoxicity compared with ZnCl_2_ in contrast to CoO and CuO NPs. However, more studies are warranted to evaluate the exact mechanism of these differential toxic effects between ZnO NP and ZnCl_2_.

In this study, neither fast-dissolving NPs nor metal chlorides stimulated the production of IL-1β, while biopersistent Co_3_O_4_ and TiO_2_ NPs stimulated IL-1β release with good dose-dependency. Previous studies have shown that several types of NPs (e.g., TiO_2_, SiO_2_, and Ag) as well as crystalline silica simulate IL-1β release by inflammasome activation, which can be triggered by the phagocytosis-related events including NP uptake and phagosomal leakage or endoplasmic reticulum stress (Yazdi et al., 2010; Sandberg et al., 2012; Simard et al., 2015). Thus, no significant changes in IL-1β concentration by fast-dissolving NPs and their metal chlorides imply that those substances cannot stimulate inflammasome activation, which might be due to the fast dissolution of NPs inside lysosomes (Cho et al., 2013a). The minimal changes of IL-8 by both NPs and metal chlorides observed in this study might imply that these materials does not stimulate IL-8 release at sub-lethal doses, but our previous study showed that the lethal dose of ZnO and CuO NPs can stimulate IL-8 release in A549 cells (Cho et al., 2013a).

## Conclusion

The comparison of cytotoxicity caused by fast-dissolving NPs and their constituent metal ions showed both similarities and differences. Successful development of STR for fast-dissolving NPs need precise calculation of the intrinsic toxic potential of its constituent metal ions and evaluation of similarity of dose-response curve between NP and metal ion. In this perspective, this result shows that CoO and CuO NPs can be simply predicted by its toxic potential of Co and Cu ion but ZnO NPs need more parameters for the prediction which warrant further studies. This may be mainly due to the differential mechanism of intracellular uptake of these substances and their interaction with detoxification molecules such as metallothionein.

## Author Contributions

Participated in the experimental design and data analysis: JJ, S-HK, and W-SC. Conducted the experiments: JJ and S-HK, with help of SL, D-KL, YH, and SJ. Wrote the first draft of the manuscript: JJ and S-HK. Contributed to the writing of the manuscript: SL, D-KL, YH, SJ, and W-SC. All authors read and approved the final version of the manuscript.

## Conflict of Interest Statement

The authors declare that the research was conducted in the absence of any commercial or financial relationships that could be construed as a potential conflict of interest.
